# Simultaneous determination of 11 kinds of siloxanes in drinking water and source water using solid-phase microextraction combined with gas chromatography-mass spectrometry

**DOI:** 10.3389/fchem.2025.1554453

**Published:** 2025-02-12

**Authors:** Sijie Liu, Jie Ma, Su Jiang

**Affiliations:** Department of Chemistry and Physics, Jilin Provincial Center for Disease Control and Prevention, Changchun, China

**Keywords:** drinking water, gas chromatography-mass spectrometry, internal standard method, siloxanes, solid-phase microextraction

## Abstract

**Background:**

Siloxanes have been widely used in various products and have been detected to varying degrees in the environment. It has been reported that methylsiloxane has toxic effects on the nervous, immune and reproductive systems of aquatic animals, and is carcinogenic and mutagenic. Therefore, in order to protect human health, it is urgent to establish a method for the determination of siloxane content in drinking water and source water.

**Methods:**

Herein, this paper proposes a precise, fast, and selective method using solid-phase microextraction combined with gas chromatography-mass spectrometry (GC-MS/MS) method for the simultaneous detection of 11 kinds of siloxanes in drinking water and source water. Quantification of siloxanes was carried out by internal standard method. The parameters that affect the extraction and desorption processes were optmised. The extraction efficiency of four commercially available SPME fibers was evaluated. The results showed that divinylbenzene/polydimethyl-siloxane or divinylbenzene/Carboxen/polydimethylsiloxane was the best coating for the extraction of siloxane.

**Results:**

This method provided good linearity (*r* > 0.9946) and precision (RSD% <8.0%) with the minimum detection mass concentration ranging from 0.008 to 0.025 μg/L under the optimized extraction and GC-MS/MS analysis conditions. The developed method has been applied to the simultaneous analysis of 11 kinds of siloxanes in drinking water and source water, and the results showed that decamethylcyclopentasiloxane (D5) and dodecamethylcyclohexasiloxane (D6) were found in two source water samples at concentrations ranging from 0.008 to 0.012 μg/L and 0.015–0.019 μg/L, respectively.

**Conclusion:**

This developed method was simple, quick, and effective and our satisfactory results demonstrated its suitability for the simultaneous determination of 11 kinds of siloxanes in drinking water and source water.

## 1 Introduction

Siloxanes are a type of synthetic, high molecular weight organosilicon polymer bearing silico-oxygen bonds (-Si-O-Si-) in their backbone with organic groups connected to the silicon atoms. Due to the volatility of its oligomers, the silicon and oxygen atoms appear alternately in a synthetic ring structure, named cyclic volatile methylsiloxane (cVMS) by the International Chemical Organization, which may break in the environment to form linear methylsiloxane (Capela et al., 2017).

Recently, siloxanes have been widely used in various products encompassing industrial, household, and personal care products, cleaning agents, and medical devices (Homem et al., 2017; Lassen et al., 2005; Graiver et al., 2003; Horii and Kannan, 2008; Genualdi et al., 2011; Lu et al., 2011). In 2023, China was expected to place into operation new capacity plans to achieve 582,000 tons/year of polysiloxane with an annual production capacity of 2.893 million tons/year, an increase of 25.2%, which will be the world’s largest supply and demand market (Zhong, 2023).

During their use, ∼100% of the volatile siloxanes enter the surrounding environment, of which 90% is distributed in the atmosphere, indoor dust, soil, and other media through volatilization and settlement. The other 10% is discharged with domestic sewage and enters environmental water bodies through surface run-off and other pathways. Consequently, siloxanes have been detected to varying degrees in the environment (Kierkegaard and McLachlan, 2010; Wang et al., 2001; Hodgson et al., 2003; Kierkegaard et al., 2013; Genualdi et al., 2011; Raich-Montiu et al., 2014; Xu et al., 2013; Guo et al., 2019; Zhang et al., 2024). In 2005, Kaj et al. (2005) reported the content of cVMS in various water environments in Northern Europe (including natural surface water bodies, seawater, stormwater run-off, water inlet, and outlets from municipal sewage treatment plants, landfill leachate, etc.). Although there are some defects in the detection method and technology, these results indicate the future direction for the detection of cVMS in the environment. Wang et al. (2013b) investigated the content of cVMS in wastewater (wastewater inlets and outlets, and receiving surface water) of a wastewater treatment plant in Canada, and the concentrations of octamethylcyclotetrasiloxane (D4), decamethylcyclopentasiloxane (D5), and dodecamethylcyclohexasiloxane (D6) in wastewater effluent were 6.69, 135 and 26.9 μg/L, respectively. The highest detected amounts of D4, D5, and D6 in surface water ranging from 0.005 to 3.1 km away from the sewage outlet were 0.023, 1.48, and 0.151 μg/L, respectively. It has been reported that methylsiloxane has toxic effects on the nervous, immune and reproductive systems of aquatic animals, and is carcinogenic and mutagenic. However, there are few research studies on siloxanes in drinking water and there is no limiting standard for siloxanes. Meanwhile, several methods, including headspace, liquid-liquid microextraction, and purge/capture techniques combined with gas chromatography-mass spectrometry analysis have been developed for the determination of siloxanes in solid and natural water samples (Companioni-Damas et al., 2012; Wang et al., 2023; Luciana et al., 2018; Sparham et al., 2008; Cortada et al., 2014; Sparham et al., 2011; Zhang et al., 2011; Wang et al., 2013a). However, Previous studies normally require a sample preparation step because organic compounds are contained at trace levels or in a matrix that interferes. Solid phase microextraction (SPME)has attracted much attention because it has the advantages of integrating sampling, extraction, concentration and sampling, and the need for solvent-free micro-extraction of samples. Headspace-solid phase microextraction combined with gas chromatography-mass spectrometry technology has the advantages of rapidity, sensitivity, accuracy and high efficiency. Organic compounds with different concentrations and chemical structures can be analyzed. The target compound loss rate in the sample is low, which is suitable for the analysis of multi-component mixture samples, and has been used as the first choice for the determination of siloxane in recent years. Xu et al. (2013) reported that determination of 6 volatile siloxanes in Water by headspace solid phase microextraction/Gas chromatogram-mass spectrometry. The detection limit of this method is about 5 ng/L, and its sensitivity and precision can meet the requirements of water quality detection. The DB-5MS chromatographic column has been used for the separation and detection. However, there are some problems in the detection method and technology of siloxanes in drinking water and source water. The DB-5MS column bleeding compound were the target compound for detection. When the column bleeding is large, it cannot accurately reflect the true content of cyclic siloxanes (D4, D5, D6, etc.) in the sample. In addition, the high volatility of siloxanes and the potential sources of background contamination affecting their final determination are the main limiting factors for their analysis (Luciana et al., 2018). Meanwhile, these methods are limited to the detection of only a few siloxanes, which fails to meet the requirements for comprehensive detection. Therefore, it is imperative to further investigate and refine the SPME experimental conditions to ensure the detection of a broader range of siloxane compounds, particularly when analyzing trace or ultra-trace levels of siloxanes in water samples.

In this study, we have established a precise, fast, and selective method using solid-phase microextraction combined with gas chromatography-mass spectrometry (GC-MS/MS) method for the simultaneous detection of 11 kinds of siloxanes in drinking water and source water. Quantification of siloxanes was carried out by internal standard method. This method was successfully applied to identification and quantification of 11 kinds of siloxanes in drinking water and source water.

## 2 Materials and methods

### 2.1 Standards, reagents, and materials

Hexamethylcyclotrisiloxane (D3), octamethyltrisiloxane (L3), octamethylcyclotetrasiloxane (D4), decamethyltetrasiloxane (L4), decamethylcyclopentasiloxane (D5), dodecamethylpentasiloxane (L5), dodecamethylcyclohexasiloxane (D6), tetradecamethylhexasiloxane (L6), tetradecamethylcycloheptasiloxane (D7), hexadecamethylcyclooctasiloxane (D8), and octadecamethylcyclononasiloxane (D9) with ≥98% purity were supplied by CATO Research Chemicals Inc. (Guangzhou. China). The internal standard, 2,4,6-triethylene-2,4,6-trimethylcyclotrisiloxane (D3V), with ≥97% purity was supplied by Macklin Biochemical Co., Ltd. (Shanghai. China).

Acetone and sodium chloride were purchased from Beijing Chemical Works. Ultrapure water (resistivity of 18.2 MΩ cm at 25°C) was obtained using a Milli-Q Gradient water system (Millipore, Bedford, MA, United States).

Stock standard solutions with concentrations of 1,000 μg/mL of the 11 kinds of siloxanes and internal standard were prepared in acetone, respectively and stored at 4°C in brown glass bottles.

### 2.2 Instrumentation

Samples were analyzed on a GCMS-QP2010 Ultra gas chromatography-mass spectrometer (Shimadzu, Japan). Chromatographic separation was conducted on a VF-WAX elastic quartz capillary column (30 m × 0.25 mm I.D. and 0.25 μm film thickness).

An solid-phase microextraction (SPME) device including SPME sampling table, SPME handle, sample catheter, and SPME fiber (using divinylbenzene/polydimethylsiloxane (DVB/PDMS) fiber or divinylbenzene/carboxen/polydimethylsiloxane (DVB/PDMS/CAR) fiber or equivalent) was used. The liner for solid phase microextraction had dimensions of 78.5 mm × 6.3 mm, 0.75 mm.

### 2.3 Sample preparation

Brown glass bottles with Teflon pads were used for sample collection. During sampling, water was collected into a full bottle, sealed, and stored at 4°C. The determination needed to be completed within 24 h because the components to be detected in the sample were volatile. A 0.22 μm water filter membrane was used to filter the water sample if the sample was turbid.

### 2.4 Solid phase extraction

A magnetic stirrer was placed into a 60-mL sampling bottle and 4 g of sodium chloride added. 40 mL of water sample were added, followed by 80 μL of the internal standard solution (100 μg/L), and the bottle cap tightened. The bottle was placed on the sampling table and stirred at 800 rpm. After 15 s of stirring, the extracted fiber was pressed to the top space for adsorption extraction. After 45 min of extraction, the extracted fiber was removed, the needle dried and the water was absorbed, and the extracted fiber inserted into the gas chromatographic inlet and desorbed at 220°C for 2 min.

### 2.5 GC-MS/MS analysis conditions

All samples were analyzed using GC-MS/MS. For GC detection, the separation was conducted on a VF-WAX elastic quartz capillary column. The oven temperature was programmed from 35°C (held for 2 min) to 240°C at 10°C/min (held for 5 min). Helium (99.999%) was used as a carrier gas at a constant flow rate of 1.0 mL/min. The injector temperature was maintained at 220°C and a 50:1 split injection mode with the split closed for 2 min used for our experiments.

**FIGURE 1:**
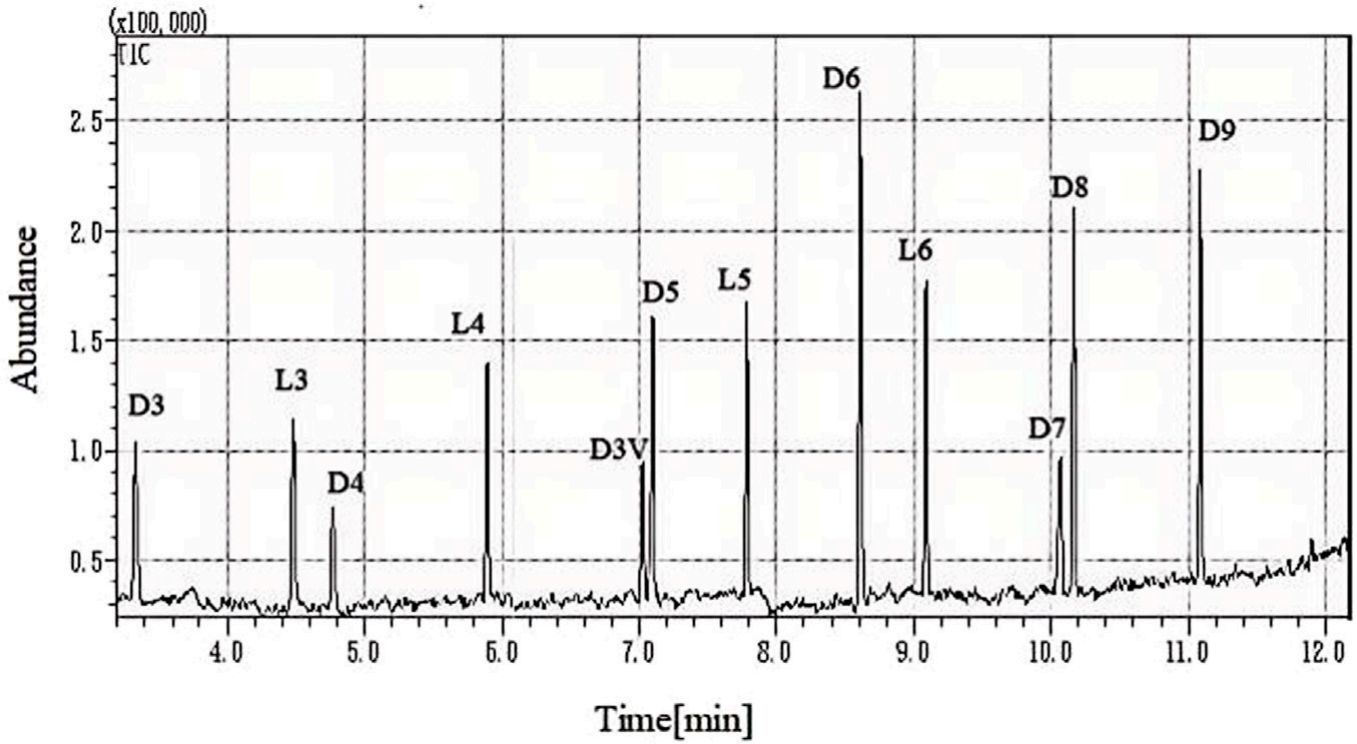
Total ion flow diagram of 11 siloxanes and internal standard solutions.

Electron ionization (EI) mode operated at 70 eV was applied for MS/MS detection. The mass spectrometer source and transfer line were set at 230°C and 280°C, respectively. Selected ion monitoring (SIM) mode was used for data acquisition. Table 1 shows the ions selected for the quantification and confirmation of 11 kinds of siloxanes using the GC-MS method. The representative total ion flow diagram of the 11 siloxanes and internal standard solutions is shown in Figure 1.

**TABLE 1:** Quantification and confirmation ions selected of 11 kinds of siloxanes.

Compound	RT (min)	Quantification ion (m/z)	Confirmation ion (m/z)
D3	2.9	207	133, 96
L3	3.6	221	205, 103
D4	4.1	281	282, 265
L4	4.5	207	295, 208
D5	5.8	267	355, 356
D3V	7.0	243	215, 203
L5	7.1	147	281, 282
D6	7.8	341	429, 325
L6	8.6	221	147, 281
D7	9.1	281	147, 327
D8	10.2	355	221, 356
D9	11.1	429	341, 147

## 3 Results and discussion

### 3.1 Optimization of the sample preparation conditions

According to the physical and chemical properties of siloxanes, three pretreatment methods for solid phase extraction, solid phase microextraction, and purge and capture were selected for comparison. The absolute recoveries of D3, D4, and L3 were <30% in the solid phase extraction method and the small ring siloxanes were highly volatile, so it was not suitable for this method. The principles of purge capture and SPME are similar. The experiment was conducted using SPME first considering the operability of the method and the availability of consumables, the molecular weight of siloxanes D3 to D9 were very different, and stirring SPME was more beneficial to the volatilization of the tested substances.

#### 3.1.1 Selection of extracted fibers

Forty milliliters the mixed standard solution (100 ng/L) were added to a 60-mL headspace bottle. Under the extraction conditions of room temperature, pH 7.0, extraction time of 60 min, and desorption time at gas chromatographic inlet (220°C) of 2 min, the extraction efficiency of four kinds of fibers (polyacrylate (PA), Polyethylene glycol/divinylbenzene (CW/DVB), DVB/PDMS, and DVB/PDMS/CAR) on the 11 kinds of siloxanes was investigated. Information regarding the four kinds of fibers is shown in Table 2.

**TABLE 2:** 4 kinds of extracted fiber information.

Extracted fibre	PA	CW/DVB	PDMS/DVB	PDMS/DVB/CAR
Coating thickness, μm	85	65	65	50/30
Needle length, cm	1	1	1	1

**FIGURE 2:**
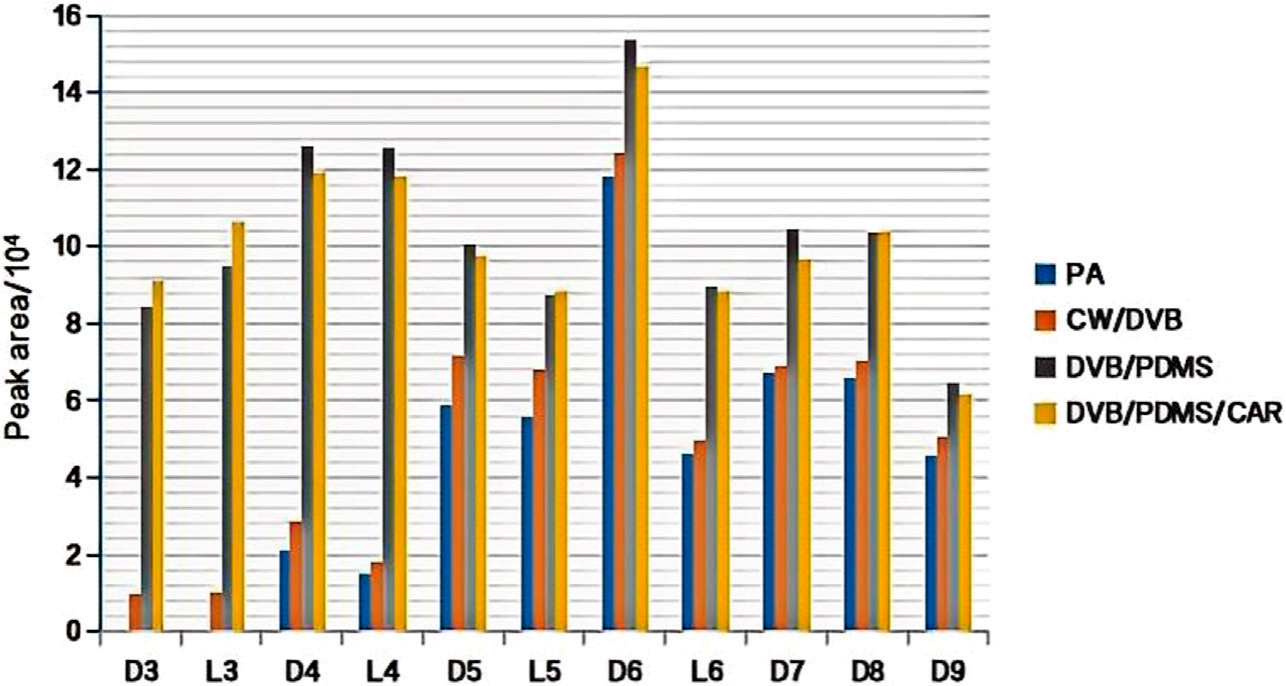
Influence of different fibers on extraction efficiency of target substance.

Our results showed that the extraction efficiency of the DVB/PDMS and DVB/PDMS/CAR fibers for the 11 kinds of siloxanes was significantly higher than that of the PA and CW/DVB fibers, which may be attributed to the main component of DVB/PDMS and DVB/PDMS/CAR being weakly polar methicone polymer (PDMS). Consequently, the DVB/PDMS and DVB/PDMS/CAR fibers exhibited a higher extraction efficiency than the polar PA and CW/DVB fibers. DVB/PDMS and DVB/PDMS/CAR had similar extraction efficiencies for D4, L4, D5, L5, D6, L6, D7, D8. and D9. For D3 and L3, the extraction efficiency of DVB/PDMS/CAR was slightly higher than that of DVB/PDMS. However, the reproducibility (RSD of 2.6%–7.8%) of DVB/PDMS extraction was better than that observed for the 11 kinds of siloxanes using DVB/PDMS/CAR (RSD of 5.6%–17.4%), as shown in Figure 2.

After a complete SPME/GC-MS process of the 500 ng/L target solution, the two fibers were directly inserted into the chromatographic inlet for secondary desorption. Our results showed that the peak area of the 11 kinds of siloxanes during the second desorption of DVB/PDMS was 0.5% lower than that of the first desorption, while the peak area of the target siloxanes during the second desorption of DVB/PDMS/CAR was ∼1% of that of the first desorption. Although the absolute recovery of DVB/PDMS fiber was slightly better than that of DVB/PDMS/CAR fiber in the extraction of the target materials, there was no significant difference when applying the internal standard method to determination. Based on the reproducibility and residue of the two fibers, the DVB/PDMS and DVB/PDMS/CAR fibers were used in this experiment.

#### 3.1.2 Selection of the extraction temperature

**FIGURE 3:**
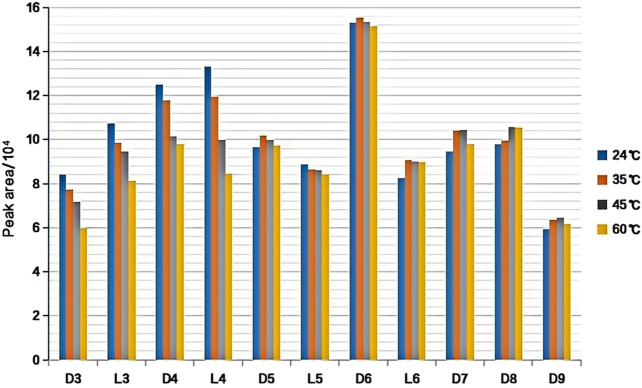
Effect of extraction temperature on extraction efficiency of target substance.

Under the following experimental conditions, DVB/PDMS fiber, 40 mL of the standard solution (100 ng/L) was mixed with the 11 kinds of siloxanes added to a 60-mL headspace bottle, pH 7.0, extraction time of 60 min, and desorption for 2 min at 220°C at the gas chromatography inlet. The effect of the extraction temperature on the extraction efficiency was investigated. Figure 3 shows that the extraction efficiency decreased with an increase in the extraction temperature, and the extraction efficiency of L3, L4, D3, and D4 reached its maximum value at 24°C (room temperature). Our results indicated that the extraction efficiency of L3, L4, D3, and D4 could be satisfactory at room temperature due to their high volatility, but the extraction efficiency was not significantly improved upon heating. On the contrary, high temperature reduced the partition coefficient between the fiber coating and top space, resulting in a reduction in the extraction efficiency (peak area). D5, L5, D6, and L6 exhibited the highest extraction efficiency at 35°C, while D7, D8, and D9 had the highest extraction efficiency at 45°C. Room temperature (24°C) was chosen as the optimal extraction temperature when considering the main polluents in the environment are D3, D4, D5, and D6.

#### 3.1.3 Selection of the extraction time

**FIGURE 4:**
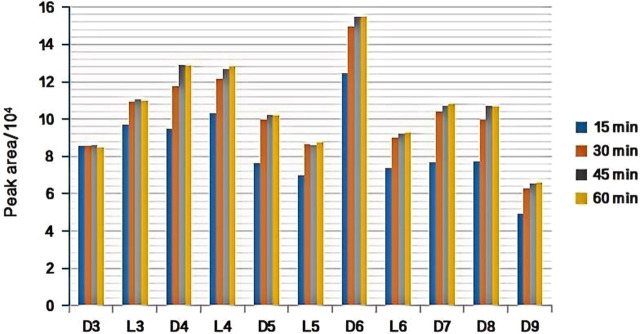
Effect of extraction time on extraction efficiency of target substance.

Under the following experimental conditions of DVB/PDMS fiber, 40 mL of the standard solution (100 ng/L) was mixed with the 11 kind of siloxanes added to a 60-mL headspace bottle, pH 7.0, extraction temperature of 24°C, and desorption for 2 min at 220°C at the gas chromatography inlet. It can be seen from our experimental results that with an increase in the extraction time, the extraction efficiency of the 11 kinds of siloxanes increased. When the extraction time reached 45 min, the extraction reached equilibrium, and the peak area changed little when the extraction time reached 60 min (see Figure 4). To reduce the analysis time, 45 min was used as the optimized extraction time.

#### 3.1.4 Selection of the extraction salinity

**FIGURE 5:**
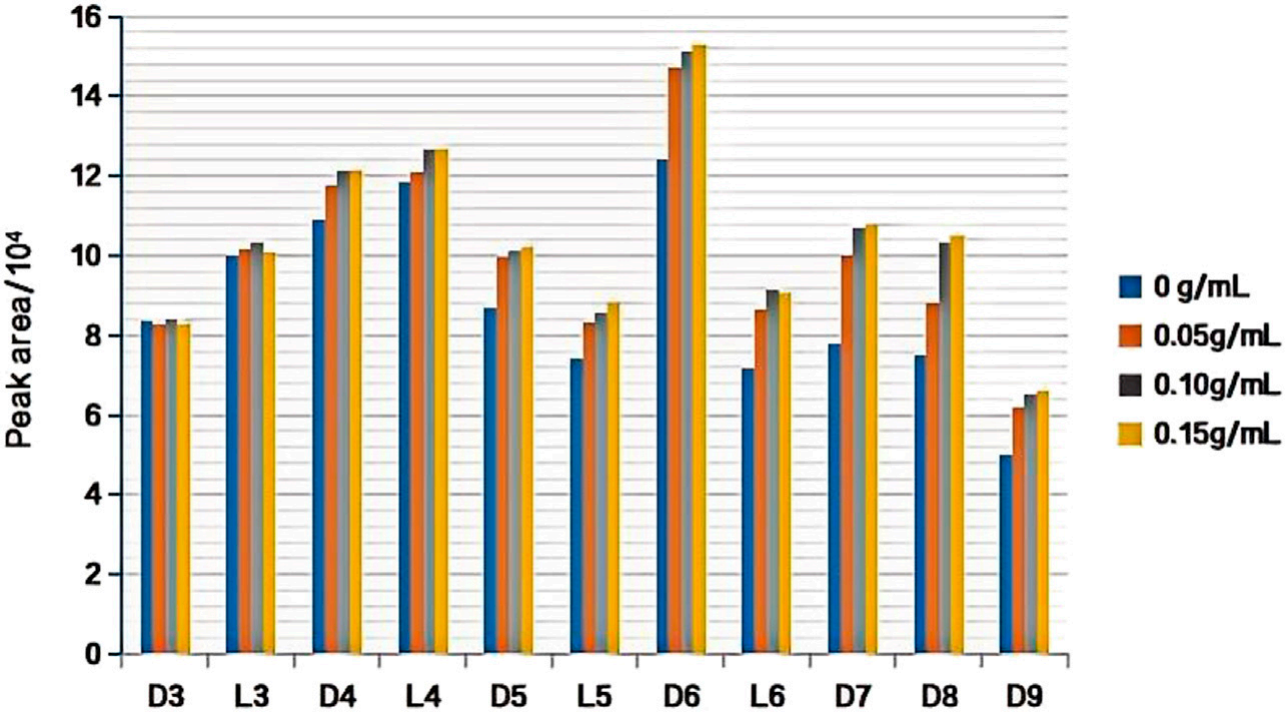
Effect of NaCl concentration on extraction efficiency of target substance.

The addition of inorganic salts (such as NaCl) to the aqueous solution induced a salting-out effect, thereby increasing the ionic strength of the solution. This effectively reduced the solubility of the analyte in the solution and enhanced its volatility towards the headspace phase. Consequently, it enabled greater absorption of the target components by the fiber coating for the purpose of analysis, which ultimately improved the extraction efficiency. The effects of salinity on the extraction efficiency were investigated by adding different concentrations of NaCl to the water samples. The experimental conditions were as follows: DVB/PDMS fiber, 40 mL of standard solution (100 ng/L) mixed with the 11 kind of siloxanes added to a 60-mL headspace bottle, pH 7.0, extraction temperature of 24°C, extraction time of 45 min, and desorption for 2 min at 220°C at the gas chromatography inlet. Our results showed that the extraction efficiency of L3 and D3 could not be significantly increased upon adding NaCl to the water samples. However, the extraction efficiency of L4, D4, D5, L5, D6, L6, D7, D8, and D9 slowly increased with an increase in the NaCl concentration and when the NaCl concentration reached 0.10 g/mL, the extraction efficiency reached its maximum value (see Figure 5).

### 3.2 Selection of the injection port desorption time

**FIGURE 6:**
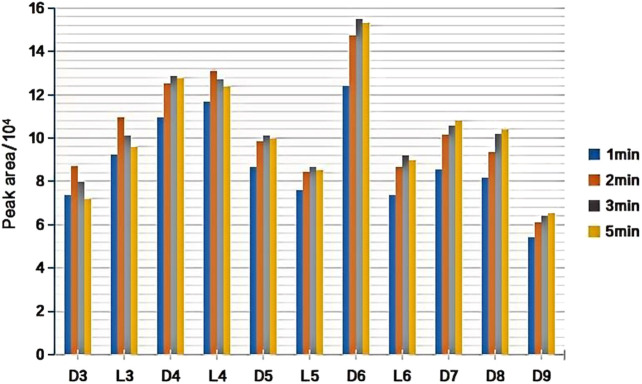
Effect of desorption time on extraction efficiency (peak area) of target substance.

The experimental conditions were as follows: DVB/PDMS fiber, 40 mL of standard solution (100 ng/L) mixed with the 11 kinds of siloxanes added to a 60-mL headspace bottle, extraction temperature of 24°C, pH 7.0, extraction time of 45 min, and desorption at 220°C at the gas chromatography inlet. The influence of the desorption time at the gas chromatography inlet on the extraction efficiency of the target substance was investigated (Figure 6). Our results showed that the extraction efficiency (peak area) of L3, D3, and L4 was largest when the desorption time was 2 min, and the extraction efficiency significantly decreased upon extending the desorption time. The extraction efficiency of D4, D5, L5, L6, and D6 was highest at 3 min of desorption and the extraction efficiency of D7, D8, and D9 was highest at 5 min of desorption.

Although D4–D6 did not reach the maximum extraction efficiency at 2 min, the difference between D4 and D6 was not obvious. Therefore, considering that the extraction efficiency of L3 and L4 was significantly reduced when the desorption time was greater than 2 min, 2 min was selected as the final optimized desorption time.

### 3.3 Optimization of the GC-MS/MS method

#### 3.3.1 Column selection

We compared the DB-5MS (30 m × 0.25 mm, 0.25 μm) elastic quartz capillary column with a VF-WAX MS (30 m × 0.25 mm, 0.25 μm) elastic quartz capillary column. The DB-5MS column bleeding compound was the target compound of detection. When the column bleeding was large, it could not accurately reflect the true content of cyclic siloxane (D3, D4, D5, D6, etc.) in the sample. The VF-WAXms column was a relatively low loss column among all of the commercially available products and had less interference on target compounds, so this column was used in our study.

#### 3.3.2 Selection of the chromatographic conditions

Because D3, D4, and L3 were extremely volatile, the initial temperature of the chromatography should be as low as possible under the premise of ensuring the complete separation of each target compound. Finally, the initial temperature was selected to be 35°C, held for 2 min, and the temperature was raised to 240°C at 10°C/min. The other conditions were as follows: Inlet temperature: 220°C; carrier gas: high purity helium; column flow rate: 1.0 mL/min; shunt injection, shunt ratio: 50:1.

#### 3.3.3 Selection of the internal standard

At present, D3V is not detected in water and air, its physical and chemical properties are similar to the target objects to be measured, and its retention time was in the middle of the 11 kinds of siloxanes studied, so it was selected as the internal standard.

### 3.4 Method evaluation

The method was assessed in terms of the detection limit, linearity range, calibration curve, precision, and accuracy using drinking and source water samples, respectively. The minimum detection mass concentration of the method was D3, 0.020 μg/L; L3, 0.012 μg/L; D4, 0.015 μg/L; L4, 0.010 μg/L; D5, 0.012 μg/L; L5, 0.020 μg/L; D6, 0.008 μg/L; L6, 0.022 μg/L; D7, 0.018 μg/L; D8, 0.018 μg/L; and D9, 0.025 μg/L. The linearity of the method was tested at six different concentration levels within the range of 0–10.0 μg/L. Each concentration level was analyzed in sextuplicate. The linearity of the calibration curve was assessed using the coefficient of determination. A good linearity was observed in the range of 0–10.0 μg/L for the 11 kinds of siloxanes. The precision and accuracy of the method were determined using spiked samples with three different levels (0.1, 0.5, and 2.0 ng/L). The accuracies of the method were in the range of 80%–98% and precision of the method was 1.8%–8.0%, respectively, indicating a compliance with the requirements of residual analysis. All of the data are summarized in Tables 3, 4.

**TABLE 3:** Analytical performance of 11 kinds of siloxanes by GC-MS/MS.

Compound	Calibration curve	Correlation coefficient (*r* ^2^)	Limit of detection (ng/L)
D3	Y = 1868.6X+2,880	0.9980	6.8
L3	Y = 1,654.4X+207	0.9983	3.7
D4	Y = 1,530.2X+4,929	0.9992	4.9
L4	Y = 1,634.6X+138	0.9991	3.5
D5	Y = 1,219.7X+1,141	0.9950	4.1
L5	Y = 1,052.3X+254	0.9993	6.9
D6	Y = 2,210.5X+3,841	0.9984	2.3
L6	Y = 1,138.2X+149	0.9972	7.4
D7	Y = 1,310.1X+1840	0.9981	6.0
D8	Y = 1,268.4X+1,343	0.9946	6.2
D9	Y = 786.9.6X+769	0.9965	8.7

**TABLE 4:** Recovery rate and relative standard deviation test (n = 6).

Compound	Sample	Background concentration (μg/L)	Spiked concentration (μg/L)	Accuracy (%)	Precision (RSD,%)
D3	Tap water	ND	0.1.0.5.2.0	81, 89, 91	4.3, 3.9, 3.2
	Pure water	ND		86, 88, 87	6.5, 3.1, 4.4
	Source water	ND		82, 90, 92	4.2, 3.9, 4.5
L3	Tap water	ND		83, 89, 90	4.4, 3.0, 2.2
	Pure water	ND		80, 84, 88	6.4, 3.7, 4.9
	Source water	ND		81, 84, 91	3.8, 3.0, 2.5
D4	Tap water	ND		85, 84, 92	5.2, 2.9, 3.5
	Pure water	ND		81, 87, 94	8.0, 6.5, 3.6
	Source water	ND		80,91,95	3.3, 3.5, 2.6
L4	Tap water	ND		85, 84, 89	5.2, 3.9, 3.1
	Pure water	ND		90, 87, 93	3.8, 3.9, 3.4
	Source water	ND		84, 87, 92	5.0, 2.6, 3.3
D5	Tap water	ND		89, 91, 93	7.1, 4.2, 4.4
	Pure water	ND		87, 88, 94	4.8, 2.9, 3.1
	Source water	ND		89, 85, 92	4.1, 3.0, 3.7
L5	Tap water	ND		81, 88, 89	6.5, 3.9, 3.5
	Pure water	ND		80, 86, 89	5.9, 3.8, 3.3
	Source water	ND		82, 90, 94	4.2, 2.9, 3.7
D6	Tap water	ND		84, 88, 93	5.3, 4.6, 3.5
	Pure water	ND		90, 92, 96	4.4, 3.7, 3.6
	Source water	ND		87, 89, 93	4.0, 3.9, 3.5
L6	Tap water	ND		84, 85, 92	3.9, 3.8, 4.1
	Pure water	ND		85, 90, 95	4.3, 3.6, 2.5
	Source water	ND		80, 84, 89	4.5, 3.8, 4.0
D7	Tap water	ND		82, 84, 91	4.7, 5.3, 2.8
	Pure water	ND		86, 92, 91	5.3, 3.8, 3.9
	Source water	ND		89, 92, 98	5.5, 5.2, 3.8
D8	Tap water	ND		87, 86, 90	4.9, 3.7, 4.4
	Pure water	ND		88, 89, 94	4.8, 4.2, 4.4
	Source water	ND		85, 92, 90	5.2, 3.9, 4.5
D9	Tap water	ND		85, 87, 88	4.8, 4.0, 1.8
	Pure water	ND		82, 85, 90	4.5, 2.5, 3.4
	Source water	ND		89, 90, 92	4.7, 5.4, 2.4

### 3.5 Sample analysis

This method was successfully applied to the identification and quantification of 11 kinds of siloxanes in 10 drinking water and 10 source water samples collected from Jilin province of China. None of the 11 substances were detected in the 10 samples of drinking water. D5 and D6 were detected in two samples of source water with D5 concentrations of 0.012 and 0.008 μg/L and D6 concentrations of 0.015 and 0.019 μg/L, respectively.

## 4 Conclusion

In this study, we have established a precise, fast, and selective method using solid-phase microextraction combined with gas chromatography-mass spectrometry (GC-MS/MS) method for the simultaneous detection of 11 types of siloxanes in drinking water and source water. Quantification of siloxanes was carried out by internal standard method. The results show that DVB/PDMS or DVB/PDMS/CAR fiber is the most effective coating for extracting siloxanes. This method provided good linearity (*r* > 0.9946) and precision (RSD%<8.0%), and low limits of quantification, from 0.008 to 0.025 μg/L. The developed method has been applied to the simultaneous analysis of 11 kinds of siloxanes in drinking water and source water, and the results showed that decamethylcyclopentasiloxane (D5) and dodecamethylcyclohexasiloxane (D6) were found in two source water samples at concentrations ranging from 0.008 to 0.012 μg/L and 0.015–0.019 μg/L, respectively.

To the best of our knowledge, this is the first report on using solid-phase microextraction combined with gas chromatography-mass spectrometry (GC-MS/MS) method for the identification and quantitation of 11 types of siloxanes in drinking water and source water. The extraction was performed using solid-phase microextraction to reduce the interference during the analysis. Meanwhile, D3V was used as the internal standard during the sample preparation step. This method only required 15 min for separation and detection, and was capable to achieve rapid qualitative detection, making it more suitable for the detection of emergent public health events. Therefore, the developed method was simple, quick, and effective and our satisfactory results demonstrated its suitability for the simultaneous determination of 11 kinds of siloxanes in drinking water and source water.

As the fillers of gas chromatography columns, methylpolysiloxane compounds including D3, D4, D5, D6, D7, D8, and D9 are commonly used and can contribute to column loss. In this study, various brands and models of columns were evaluated through extensive testing, leading to the selection of the VF-WAX column due to its minimal column loss and negligible detection of target siloxanes. PDMS/DVB and PDMS/DVB/CAR fibers contain limited amounts of siloxanes, making them suitable for detecting 11 siloxanes. However, to detect a broader range of siloxanes, future research should focus on improving methodologies and techniques. Therefore, it is imperative to further investigate and refine the solid-phase microextraction experimental conditions to ensure the detection of a broader range of siloxane compounds, particularly when analyzing trace or ultra-trace levels of siloxanes in water samples.

## Data Availability

The original contributions presented in the study are included in the article/supplementary material, further inquiries can be directed to the corresponding author.
